# Adaptation, testing, and use of the "iSupport for Dementia" program in different countries: a systematic review

**DOI:** 10.1590/1980-5764-DN-2023-0097

**Published:** 2024-08-26

**Authors:** Larissa Corrêa, Aline Cristina Martins Gratão, Déborah Oliveira, Elizabeth Joan Barham, Fabiana de Souza Orlandi, Keila Cristianne Trindade da Cruz, Ana Carolina Ottaviani, Diana Quirino Monteiro, Gustavo Carrijo Barbosa, Anabel Machado Cardoso Alvarenga Pilegis, Luana Aparecida da Rocha, Ludmyla Caroline de Souza Alves, Luiza Barros Maciel, Camila Rafael Ferreira Campos, Sofia Cristina Iost Pavarini

**Affiliations:** 1 Universidade Federal de São Carlos, Programa de Pós-Graduação em Enfermagem, São Carlos SP, Brazil.; 2 Universidade Federal de São Carlos, Departamento de Gerontologia, São Carlos SP, Brazil.; 3 Universidad Andrés Bello, Faculty of Nursing, Doctorate Programme in Nursing Science, Campus Viña del Mar, Chile.; 4 Millenium Institute for Care Research (MICARE), Santiago, Chile.; 5 Universidade Federal de São Carlos, Programa de Pós-Graduação em Psicologia, São Carlos SP, Brazil.; 6 Universidade Federal de São Carlos, Departamento de Psicologia, São Carlos SP, Brazil.; 7 Universidade de Brasília, Faculdade de Ciências da Saúde, Departamento de Enfermagem, Brasília DF, Brazil.

**Keywords:** Caregivers, Dementia, Internet-Based Interventions, Systematic Review, Cuidadores, Demência, Intervenção Baseada em Internet, Revisão Sistemática

## Abstract

The World Health Organization developed the "iSupport for Dementia" program for family caregivers of people with dementia.

**Objective:**

To explore studies on adaptation, randomized clinical trial protocols, and preliminary results of iSupport by unpaid caregivers of people living with dementia in different countries.

**Methods:**

Systematic review.

**Results:**

Ten cultural adaptation studies, eight randomized clinical trial protocols, and two preliminary results were included. Adaptation studies showed adjustments in terminology, design, and additional resources. Clinical trial protocols included burden as the primary outcome, and baseline, three months of intervention, and follow-up after six months. Studies with preliminary results found positive effects on the mental health and well-being of caregivers after using the program.

**Conclusion:**

iSupport is an online program of the World Health Organization in response to dementia in implementation in different countries.

## RESUMO

A Organização Mundial da Saúde desenvolveu o programa "iSupport for Dementia" para cuidadores familiares de pessoas com demência.

**Objetivo:**

Explorar estudos sobre adaptação, protocolos de ensaio clínico randomizado e resultados preliminares do "iSupport" por cuidadores não remunerados de pessoas que vivem com demência em diferentes países.

**Resultados:**

Foram incluídos dez estudos de adaptação cultural, oito protocolos de Ensaio Clínico Randomizado e dois resultados preliminares. Os estudos de adaptação apresentaram os ajustes nas terminologias, design e recursos adicionais. Os protocolos de ensaio clínico incluíram a sobrecarga como desfecho primário, e com linha de base, três meses de intervenção e acompanhamento após seis meses. Os estudos com resultados preliminares encontraram efeitos positivos na saúde mental e bem-estar dos cuidadores após o uso do programa.

**Conclusão:**

O iSupport é um programa online da Organização Mundial da Saúde em resposta à demência na implantação em diferentes países.

## INTRODUCTION

There is a growing trend toward the use of technological health interventions, such as online programs, especially in low- and middle-income countries^1^. Examples of this intervention model are mobile phone storytelling app^2^, remotely supervised online chair yoga, and visual socialization^3^. According to a review study, the most common are telehealth (interaction via the web) and telemedicine (real-time interaction)^4^.

This is partly due to the massive use of mobile devices and the population's growing access to the Internet, even in geographically isolated regions, as well as the fact that they can have many advantages for health services, such as greater adaptability, greater reach, and lower cost^5,6^.

Online interventions gained even increased prominence following the COVID-19 pandemic, a moment that required social isolation and the search for digital solutions^7^. Furthermore, interventions that meet the demands of informal caregivers of people with dementia (PwD) are recommended in the literature, so that they can be carried out with greater flexibility and adapted individually, improving resilience^8^, and decreasing social isolation and loneliness^9^.

Online interventions can increase adherence to the program because the caregivers do not need to commute and can easily integrate participation into the care routine^10,11^. In addition, various studies show positive effects of such interventions on feelings of anxiety, overload, depression, stress, well-being, and quality of life^12–16^.

Given the range of advantages of online disciplines, and the urgent need for training disciplines and strengthening support in an accessible, acceptable, and effective way for informal caregivers of PwD, a public health response priority was created through the Global Action Plan 2017–2025^17^. To this end, a program known as "iSupport for Dementia" was developed by the World Health Organization (WHO).

iSupport is a program aimed at meeting international goals to support families or other unpaid people (like friends and neighbors) affected by dementia and the need to formulate technological strategies to solve global problems. It is an online support and training platform for family caregivers of PwD. The program is in the phase of adaptation, testing, and implementation process in more than 20 countries, such as Portugal, India, Australia, China, and Brazil, among others^18^.

The WHO requires that, to be used by a member country, iSupport must be culturally adapted and its effects evaluated before being made available to that population^19^. However, the search for scientific evidence should be aligned with the different contexts in which iSupport is expected to be used. This systematic review is presented as a way to identify iSupport publications and analyze the differences among them so that it can support future researches about the program, enriching this gap in evidence in order to assist in the effective implementation and use of iSupport around the world. Therefore, the objective of this systematic review was to explore the existing studies on the adaptation, (randomized clinical trial) RCT protocols, and the preliminary results of this RCT of the iSupport for Dementia program for use by unpaid caregivers of PwD in different countries.

## METHODS

The review was conducted following the Preferred Reporting Items for Systematic Review and Meta-Analysis (PRISMA)^20^, and its protocol was previously registered on the International Prospective Register of Systematic Reviews (PROSPERO) platform under CRD42022343168. Searches were carried out in February 2023 in the following databases: Virtual Health Library (VHL), PubMed, EMBASE, and Scopus, with a new search in February 2024. For both searches, a strategy was adapted to the requirements of each database, including descriptors related to the following concepts: "family caregivers"; "dementia"; "online intervention", and "study designs" (Supplementary Material 1 – https://www.demneuropsy.com.br/wp-content/uploads/2024/04/DN-2023.0097-Supplementary-Material.docx). The reason for these different strategies on databases is intended to meet the different specifications of each base.

The inclusion criteria for the study were:

- Evaluated elements of cross-cultural adaptation, usability, and/or effectiveness;
- Online interventions from the iSupport for Dementia program;
- Family caregivers of PwC destination;
- Publications in English; and
- Qualitative or quantitative designs.

The following were excluded:

- iSupport use for another public;
- Anyone Internet-based intervention than not iSupport; and
- Conference abstracts that were not linked to a full-text publication, literature reviews, editorials, letters to the editor, expert interviews, study protocols, positioning papers, dissertations, theses, books, and opinion papers.

The selected documents were exported to the Zotero® software, in which duplicates were identified and excluded. Three researchers (ACO, DQM, and LC) independently examined and extracted the data from the studies. Those that met the eligibility criteria were read in full by the same researchers. Any disagreements regarding the inclusion or exclusion of each article were resolved in a meeting with all authors of this review. Seeking to obtain as many relevant articles as possible, upon recommendation of the PRISMA statement^20^, a manual search was carried out on the reference list of the articles selected according to the eligibility criteria. The steps undertaken by the authors occurred independently to avoid selection bias^21^. All selected articles were available in full.

For data extraction, a protocol was previously elaborated with determinate variables to be examined at each type of study. Charts were then created to be completed according to study types. For adaptation, the following were extracted:

- Study identification (authors, year, and country of publication);
- Design;
- Sample size;
- Method; and
- Main findings (process stages and main changes in content).

The charts about RCT protocols and RCT preliminary results present the same data:

- Study identification (authors, year, and country of publication);
- Design;
- Sample size;
- Primary outcome;
- Secondary outcome;
- Monitoring period (pre-test, post-test, follow-up, and recruitment time);
- Control group (if any, and what type of intervention).

**Figure 1:**
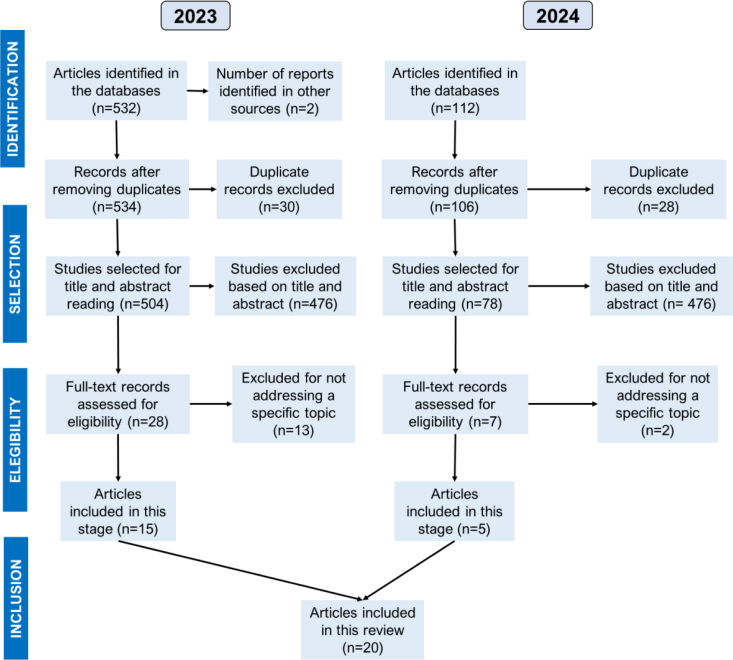
Preferred Reporting Items for Systematic Review and Meta-Analysis (PRISMA) flowchart.

Figure 1 shows the PRISMA flowchart corresponding to the selection process for this review. A total of five studies were included.

Qualitative analysis of adaptation studies was conducted regarding compliance with the guidelines made available by the WHO to licensed countries. The procedures must follow:

- Authorization by the WHO regarding the cultural adaptation of the iSupport program;
- Translation of program content;
- Cultural adaptation of iSupport content through focus groups and with the participation of researchers government representatives; and
- Verification of fidelity by the WHO^11^.

**Figure 2:**
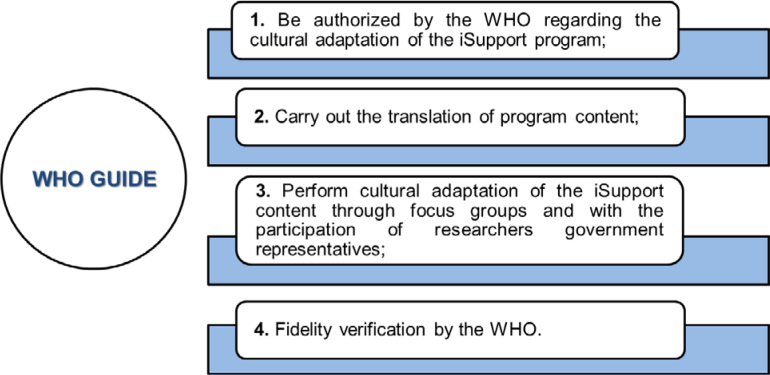
Procedures necessary for the cultural adaptation of the iSupport program based on the guide provided by the WHO^19^.

Figure 2 illustrates the process recommended by the WHO.

The preliminary results of studies were also evaluated for methodological quality, however, using the Joanna Briggs Institute (JBI) RCT checklist^22^. This checklist evaluates the following aspects of the studies:

- True randomization of participants;
- Hiding of assignment to groups;
- Similarity among groups at baseline;
- Blinding of participants to treatment assignment;
- Blinding of those responsible for administering the treatment;
- Blinding of outcome assessors;
- Equal treatment among groups, except for the type of intervention;
- Complete monitoring;
- Analysis of participants in the groups in which they were randomized;
- Results measured in the same way among groups;
- Reliable results measuring;
- Use of appropriate statistical analysis;
- Counting of appropriate study design and any deviations from the standard RCT drawing in the conduct and analysis of the trial.

To complete the charts referring to quality assessments, based both on the WHO guide and on the JBI checklist, a task stage was considered completed with "yes", not completed with "no", and not specified with "unclear".

Since this platform does not include guidelines for classifying quality levels, three evaluation categories were adopted based on the percentage of items with "yes" answers: low, medium, and high quality. Low quality was attributed to studies that did not answer "yes" to at least 50% of the items; medium quality, when "yes" was given from 50 to 75% of the items, and high quality, when "yes" was given to more than 75% of the items^23^. No studies were excluded based on this assessment.

## RESULTS

In 2023, 15 studies published between 2018 and 2023 were included, and in February 2024, five more, totaling 20 studies. Of these, ten were about cross-cultural adaptation and/or usability, eight were RCT protocols, and two were preliminary RCT data. The majority was published by countries from Europe (n=09), Ásia (n=5), South America (n=3), more than one continent (n=2), and in Oceania (n=1).

### Cross-cultural adaptation and usability evaluation studies

The main characteristics of the cross-cultural adaptation methodological studies are presented in Supplementary Material 2 – Chart 1 (https://www.demneuropsy.com.br/wp-content/uploads/2024/04/DN-2023.0097-Supplementary-Material-2.docx). These studies were carried out in Indonesia^24^, Spain^25^, Switzerland^26^, United Kingdom^27^, Brazil^28,29^, India^30^, Greece^31^, Portugal^32^, and Australia and China^33^. The sample sizes varied from 9^26^ to 35 participants^33^. The objectives were to describe the cross-cultural adaptation process^25–27,29–31^, to report on the results and lessons learned from the translation and adaptation^24^, to evaluate the usability and acceptability of the program^28,32^, as well as to understand perspectives on cross-cultural adaptation of iSupport, and to explore the factors that may affect its implementation^33^.

Regarding the design of this type of study, it has a predominance of mixed methodology (n=6)^24–26,28,32,33^, and the other half is qualitative (n=5)^27,29–31^. As for the sample size, it seems to be small, with a maximum of 35 participants^33^.

Although the description of the method changes among studies, they employed the same method available in a guide prepared by the WHO for member countries, with the stages required to carry out the cross-cultural adaptation of the program: assessment of the needs; translation of the content; cross-cultural adaptation; independent assessment of the content by a panel of experts, and content reliability verification by the authors of the program^24–26,29–31^. The samples included family caregivers of PwD aged at least 18 years^27,28–32^.

The main findings of cross-cultural adaptations referred to improving the content with: adjustments to terminologies, words, expressions, or even traditions^24–27,29–31,33^; suggestions regarding the platform design, as choosing the color of the letters or even adapting age-appropriate images^25,27,28,33^; and inclusion of additional resources, such as providing professionals to meet the users’ demands, relaxation audios, glossary, or referencing of relevant information, and guidance sites^25,26,30,33^. They also suggested future research studies that should identify the impact of the platform on care variables, digital skills and eHealth literacy^31,32^; improvement of the platform through more interactive sessions, videos, audios, and forums; the option to leave satisfaction comments; availability on different devices, such as computers, tablets, smartphones, and the like; the importance and need for the participation of local Alzheimer's associations in focus groups^28,29^; and establishment of support groups and professional facilitators^33^.

The quality analysis of these studies based on the WHO guide identified that the studies followed the recommendations, nine of which clearly completed all steps.

The study of Indonesia, Australia and China, Greece, Portugal, and India addressed the existence of the steps in the guide and further detailed them; however, the issue of receiving authorization from the WHO to do so is unclear. The United Kingdom does not demonstrate specific data about its usability and acceptability.

### Randomized clinical trial protocols

Such studies are presented in Supplementary Material 2 – Chart 2. They were published by researchers from Great Britain^34^, Netherlands^35^, Australia^36^, Brazil^37^, India^38^, Portugal^39^, and Japan^40^, and a study was developed in collaboration between Australia, Indonesia, New Zealand, and Vietnam^41^. Data had a variance between the years 2018^38^ and 2023^37^, however, half of the studies were developed predominantly in 2022 (n=3)^34,36,40^.

The designs included two-arm blind RCTs^34,35,41^, a two-arm RCT^38–40^, cross-method triangulation RCT in conjunction with a qualitative study^36^, and one study two-arm mixed-methods RCT^37^.

The sample size varied between 104^40^ and 390^37^ participants, with a large discrepancy between them.

The primary outcomes were burden^34,37–40^, depressive symptoms^34,37,38,40^, perceived stress^34,41^, anxiety symptoms^37,38,40^, level of mastery^35,37–38^, quality of life^36,38–40^, social support^36^, symptoms associated with dementia^36^, and use of health services for caregivers^36^. In turn, the secondary outcomes were self-efficacy^35–37,39^, depression and anxiety^34,35,41^, sociodemographic issues^34,41^, use of services^34–36^, hopefulness and person-centered approaches^38,40^, memory and behavior problems^38,39^, dementia screening^38^, positive aspects of caregiving^39^, health-related quality of life^40^, client satisfaction^40^, caregiving self-efficacy, and social support^36^.

The study developed in Brazil further intended to investigate quality of life; care domain; behavioral and memory problems related to dementia and caregivers’ reactions to these situations; PwD behaviors that the caregiver considers problematic and how to deal with them; and positive aspects of care and usability^37^.

The study period was divided into three stages: baseline, post-test after three months, and follow-up at the end of six months^34,37–41^. One of the studies completed the stages before initiating the RCT, the post-test at six months, and the follow-up at the ninth month^36^. As for the recruitment time, there was a variation of two months^36^, one year and one month^34^, one year and two months^35,36^, and two years and six months^41^; there was also a study that used recruitment through a continuous flow^37^. It is important to mention that some studies did not report recruitment time^38,39^.

Referring to the control groups, the following were selected for the intervention: educational book/e-book^34,38,39^; waiting list^35,41^; and usual care with online contents provided by local associations^36,37^. Differently, Japan chose to offer access to iSupport three months after baseline, and China and Australia made available usual care provided by the local Alzheimer's Association.

### Studies on preliminary randomized clinical trial data

The studies in this category, presented in Supplementary Material 2 – Chart 3, aimed at evaluating the efficacy and feasibility of the program and were carried out in Portugal^42^ and India^,43^. Preliminary data between the studies were from only one year: India from 2021^43^ and Portugal from 2022^42^. About the design, they were simply like RCT^43^ and two-arm mixed-methods RCT^42^, respectively.

The design follow-up period between them is different because of one datum: the study by India had one more month, but the two studies had monitoring every three months. The recruitment time is the greatest difference between them: Portugal had a recruitment time of two months^42^ and India, one year and three months^43^.

As for the sample sizes, Portugal had 44 participants and India had 55, which is no significant difference.

The burden and depressive symptoms were present in both primary outcomes^42,43^, followed by anxiety^42^.

The secondary most common outcome was self-efficacy^42,43^, with other outcomes presented individually: positive aspects of caregiving^42^, quality of life^42^, hopefulness and person-centered approaches^43^, level of mastery^43^, and self-rated health^43^.

Regarding the type of intervention for the control groups, both opted for an e-book^42,43^.

The study developed by Portugal established that its version of iSupport had good acceptability and promising preliminary results on caregivers’ mental health, knowledge, and well-being. However, a larger-scale RCT was required. It was suggested to optimize the study protocol, content, and interface of the program as well^42^.

For Baruah et al.^43^, no significant differences were identified between the intervention and control groups regarding the primary outcomes. However, for the caregivers who used the program, an improvement was seen in their attitudes toward PwD.

The methodological quality assessment of the two studies using the JBI tool classified them as medium quality, with overall scores of 61.5% (Portugal)^43^ and 69.2% (India)^42^. Scores below 100% are due to the following reasons: in the Indian study the issue of blinding for those administering the treatment did not apply; the outcome assessors were not blinded to treatment assignment; there was no complete follow-up or description and analysis of differences between groups in terms of follow-up; there was no analysis of participants in the groups to which they were randomized; and the issue of study design being appropriate and accounting for standard RCT designs did not apply.

As for the Portuguese study, the participants were not blinded to the treatment assignment; the blinding for those administering the treatment did not apply; the blinding of outcome counselors to treatment assignment was unclear; and the issue of study design being appropriate and accounting for standard RCT designs did not apply.

## DISCUSSION

It was possible to identify 20 published studies about the iSupport for Dementia program for unpaid caregivers of PwD. These studies dealt with the adaptation process, followed by RCT protocols and preliminary data on the use of the platform.

### Cross-cultural adaptation and usability evaluation studies

The studies presented high heterogeneity in the process of cross-cultural adaptation of the content and usability and acceptability evaluation of the program. They generally demonstrated satisfaction by users in using the iSupport program, as it was considered to have a friendly interface, relevant/useful content with usability rated as "excellent" and they were very satisfied with its use. It was made based on co-design, which process allowed the adaptation of several changes, mainly regarding local cultural adaptation and specific adjustments (such as grammatical and punctuation errors, and repetition of information), the improvement of interactive materials (graphics, links to local services, forums, among others), and the use of the program as a way to create a greater support network (creation of support groups, coping strategies, and the expansion of iSupport to healthcare professionals).

The importance of this co-design can be better understood through a study of a smartphone app with the collaboration of people with lived experience of dementia. This shows that the methodology brings benefits in terms of identifying and resolving usability problems during the development process. It speeds up delivery and reduces software development waste, allowing it to be adapted to the needs of the population^44^.

### Randomized clinical trial protocols

The use of varied techniques or their combination to conduct clinical trials offers special opportunities to highlight the importance of such approaches in order to achieve new discoveries and legitimize different strategies, aiming to answer research questions and expand their understanding. Thus, they enable an expansive and creative way of doing a research: inclusive, plural, and complementary^45^.

For countries with a future intention to prepare a protocol, it is important to pay attention to the quantification of participants. It will be necessary to compose the sample size and the common time requested for recruitment, thus seeking for this too.

The outcomes (primary and secondary) present great heterogeneity of variables, which can make us believe that the study's interest is based on real necessities from each country/local. Among the most common, burden and depressive symptoms are frequently mentioned by caregivers of PwD in studies, like consequences of care and with potential nocive^46^, which may justify the prominence of these variables among studies.

The most common monitoring period is of every three months after the baseline (seven studies; 87.5%), which seems to be a good option for new studies. About the interventions available for the control group, it does not have any specific to be used, even though the e-book has been more adopted. However, what does not seem to be a good choice is to allow this group to have intervention only at the end of the study.

Although the WHO recommends some guidelines for implementing iSupport, countries with preliminary data from published trials resorted to different sample sizes^36,38–40^. Therefore, samples with different numbers of participants may not support the discussion about the program effects, with the emerging need for research studies that carry out tests with more homogeneous samples.

### Studies on preliminary RCT data

Concerning the preliminary RCT data found, studies used the same design (two-arm RCT). The sample size did not have a discrepancy; however, it is important to pay attention to the conclusion made from Portugal, that it would be necessary to develop an RCT with a larger sample size.

In the same way that protocol studies outcomes were described, the primary and secondary outcomes did not follow a rule and could be assessed on demand. However, it is noted that the primary outcome with a greater prevalence was burden and depression symptoms, just as it is in protocols. The secondary outcome in this case was different from the primary, with inclusion of a greater quantity.

The monitoring period was generally up to 6 months, with a significant difference in time for recruitment among the studies.

Some difficulties were faced by countries that have already published preliminary RCT data, especially participant retention rates. Although Internet-based interventions such as iSupport have the potential to adapt to different environments, in general, there was difficulty recruiting people and keeping them until the end of the research^42,43^. Another explanation would be friction bias, which is caused when the study loses participants along its development.

Continuous engagement with stakeholders seemed to be the best way to keep people in the program, whether through calls, reminders, and partnerships with professionals who recommend using the platform^43^. Greater encouragement to use the website and the printed version had the potential to be an excellent alternative for people with less digital literacy^43^.

Therefore, unpaid caregivers of PwD need to undergo training that helps them develop skills related to the care and management of their own physical and mental health. Online interventions for caregivers of PwD need to be accessible and flexible, as the participants can adapt access to the content to their routine, according to care demands. However, these interventions must still be widely disseminated and encouraged^34^.

Some limitations were found, such as research data heterogeneity (sample sizes, mixed samples, different study designs, different recruitment times), which hindered comparisons between them. The analysis of the causes was not possible to be carried out. Quality analyses were performed based on the JBI checklist in preliminary results studies. In relation to the criteria recommended by the WHO, all studies were evaluated demonstrating high methodological quality.

However, the data from this review are innovative and may inform future research studies involving countries that are adapting the iSupport for Dementia program to their cultures and are developing support policies for unpaid caregivers of PwD, in addition to increasing intercultural communication among the countries that adopted this program.

In conclusion, this systematic review aimed at exploring existing studies on the adaptation, RCT protocols, and the preliminary results of the RCT of the iSupport for Dementia program for use by informal caregivers of PwD in different countries. Research studies were found that test and evaluate iSupport and its impact on caregivers of PwD, through different methods, designs, and recruitment methods. It is important to identify and categorize these findings, as they can contribute to other countries interested in using the program for their population and in advancing research that can benefit this audience.
